# Platypnea-Orthodeoxia Syndrome Presenting With Secondary Erythrocytosis

**DOI:** 10.7759/cureus.109143

**Published:** 2026-05-18

**Authors:** Niket Shah, Ujwala Koduru, Sarah Gergis, Kirolos Gergis, Adam Bowen, Borys Hrinczenko

**Affiliations:** 1 Internal Medicine, University of Michigan Health - Sparrow Hospital, Lansing, USA; 2 Hematology/Oncology, Michigan State University College of Human Medicine, Lansing, USA; 3 Hematology/Oncology, Michigan State University College of Human Medicine, Lansing, USA, Lansing, USA; 4 Pulmonary and Critical Care, Henry Ford Warren Hospital, Warren, USA; 5 Internal Medicine, McLaren Greater Lansing, Michigan State University, Lansing, USA; 6 Oncology, Michigan State University, Lansing, USA

**Keywords:** atrial septal defect, intracardiac shunt, patent foramen ovale, platypnea-orthodeoxia syndrome, polycythemia vera, positional hypoxemia, secondary erythrocytosis

## Abstract

Platypnea-orthodeoxia syndrome (POS) is a rare condition characterized by positional dyspnea and hypoxemia that may cause secondary erythrocytosis through chronic hypoxic stimulation. We describe a 71-year-old woman with an 80-pack-year smoking history who presented with persistent erythrocytosis and progressive positional dyspnea. Workup revealed an atrial septal defect with bidirectional shunting and normal erythropoietin, consistent with secondary erythrocytosis from suspected POS rather than polycythemia vera. In patients with unexplained erythrocytosis and positional dyspnea, evaluation for intracardiac shunting should be considered. Percutaneous closure is an effective treatment when the etiology is cardiac.

## Introduction

Platypnea-orthodeoxia syndrome (POS) is defined by dyspnea (platypnea) and arterial desaturation (orthodeoxia) that worsen in the upright position and improve with recumbency [1]. The condition results from right-to-left shunting that becomes hemodynamically significant with positional changes [2]. Intracardiac communication is the most common etiology, occurring in 87% of published cases [1]. Patent foramen ovale is more frequently implicated than atrial septal defect [1]. Despite increasing recognition, POS remains underdiagnosed [2]. Patent foramen ovale is present in approximately 25% of the general population, suggesting that the anatomic substrate for POS is far more common than the syndrome itself [1,3].

The differential diagnosis of erythrocytosis includes primary causes, such as polycythemia vera, and secondary causes, including hypoxia-driven erythropoietin elevation [4]. Right-to-left cardiopulmonary shunting is a recognized cause of secondary erythrocytosis [4].

The objective of this case report is to describe a patient referred for erythrocytosis who was suspected to have POS secondary to an atrial septal defect, illustrating how hematologic manifestations may precede recognition of the underlying cardiac pathology.

## Case presentation

A 71-year-old Caucasian woman was referred to hematology for evaluation of elevated hemoglobin. Her history included significant tobacco exposure with an 80-pack-year smoking history and cessation approximately 35 years prior, recently diagnosed hypertension, and childhood asthma.

She reported progressive exertional dyspnea over several months that had limited her activities. Notably, her symptoms had a positional component. She also reported relief with postural maneuvers.

On examination, the patient had peripheral findings suggestive of hypoxemia: erythema of the palms and soles, as well as cyanosis of the great and middle toes. She had unexplained weight loss.

**Figure 1:**
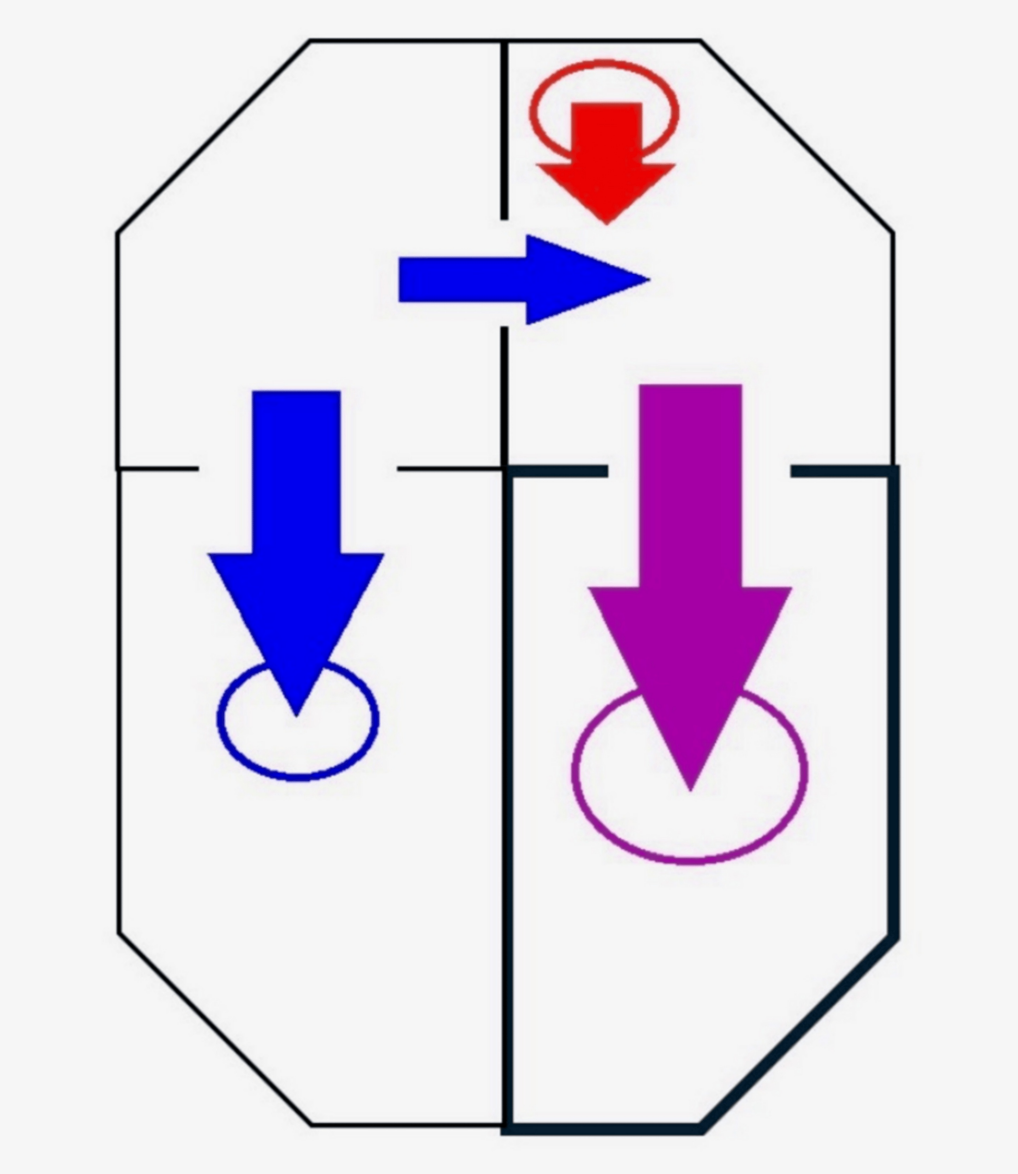
Schematic of right-to-left shunting through an atrial septal defect Blue arrows represent deoxygenated blood, the red arrow represents oxygenated blood from the pulmonary veins, and the purple arrow represents mixed blood with reduced oxygen content entering the systemic circulation.

Serial complete blood counts over two years demonstrated persistent mild erythrocytosis (Table 1). The patient's hemoglobin exceeded the WHO 2016 threshold for female erythrocytosis [4]. Workup for polycythemia vera was unrevealing. Transesophageal echocardiography (TEE) confirmed a secundum atrial septal defect with left-to-right flow and evidence of right-to-left shunting upon provocation, consistent with the physiology of platypnea-orthodeoxia syndrome (Figure 1). The erythropoietin level was within normal limits. Inflammatory markers were normal. The patient denied symptoms associated with myeloproliferative neoplasms.

**Table 1:** Serial complete blood count results Reference ranges are based on standard institutional values for adult females.

Timepoint	Hemoglobin (g/dL)	Hematocrit (%)
Reference Range (Female)	12.0 – 16.0	36.0 – 48.0
20 months prior	15.2	49.7
12 months prior	15.4	48.5
Five months prior	15.1	48.8
One month prior	16.6	50.2
At presentation	15.5	47.1

TEE confirmed a secundum atrial septal defect with left-to-right flow and preserved biventricular function. Coronary CT angiography excluded significant obstructive disease. CT angiography of the chest was negative for pulmonary embolism. Pulmonary function testing revealed an isolated reduction in FEV1 (forced expiratory volume in 1 second)/FVC (forced vital capacity) ratio with preserved FEV1, FVC, lung volumes, and diffusion capacity, consistent with mild obstruction without restrictive disease. The erythropoietin level warrants careful interpretation. In secondary erythrocytosis from hypoxia, EPO is expected to be elevated [4]. The patient's normal EPO of 8.16 mIU/mL argues against polycythemia vera, in which EPO is characteristically suppressed [5]. A truly normal EPO in the setting of erythrocytosis and suspected hypoxemia may represent an inadequate compensatory response, but still excludes autonomous erythropoiesis. The hematology assessment thus favored secondary erythrocytosis.

The patient is undergoing evaluation for percutaneous atrial septal defect (ASD) closure. To formally document orthodeoxia and characterize the degree of hypoxemia, additional testing was arranged, including ambulatory pulse oximetry with positional measurements, arterial blood gas analysis, and a six-minute walk test with continuous oximetry. From a hematologic standpoint, no cytoreductive therapy or phlebotomy was initiated, as there is no definitive evidence that the risk of thromboembolism is increased in patients with secondary erythrocytosis, and phlebotomy is not recommended routinely [4]. Diagnostic evaluation utilized the WHO 2016 criteria for polycythemia vera [4,5].

## Discussion

This case illustrates several clinically important points regarding the intersection of cardiopulmonary and hematologic disease.

Secondary erythrocytosis may be the presenting manifestation that leads to the diagnosis of POS. Our patient was referred for evaluation of elevated hemoglobin, not cardiopulmonary symptoms. Chronic intermittent hypoxemia from positional right-to-left shunting stimulated compensatory erythropoiesis. While classic descriptions of POS emphasize positional dyspnea and cyanosis, patients with chronic compensatory erythrocytosis may come to medical attention through abnormal blood counts before the underlying shunt physiology is recognized.

The workup of unexplained erythrocytosis should include consideration of cardiopulmonary causes, particularly when accompanied by dyspnea. Initial tests to differentiate primary from secondary erythrocytosis include a complete blood count, peripheral blood film, renal and liver function tests, and determination of the ferritin level [4]. When EPO is normal or elevated, secondary causes, including hypoxia, must be evaluated. The 2016 WHO diagnostic criteria for polycythemia vera require exclusion of secondary causes [5]. In patients with clinical features suggesting hypoxemia, even if resting oxygen saturation appears normal, evaluation for intermittent or positional desaturation is warranted.

The positional nature of symptoms is the critical diagnostic clue. POS should be suspected in any patient with dyspnea that worsens in the upright position and improves with recumbency [2]. The characteristic positional pattern, with dyspnea that worsens within seconds of assuming an upright position, should prompt consideration of intracardiac shunting. In cardiac POS, shunting requires both an anatomic defect (ASD or PFO) and a functional component that redirects blood flow or transiently reverses the interatrial pressure gradient [2]. Transesophageal echocardiography with bubble study is the preferred diagnostic modality [2]. An intravenous agitated bubble study should be done to assess for right-to-left shunting, with the test considered positive if bubbles appear within the left atrium within three cardiac cycles [2].

Percutaneous closure offers excellent outcomes when the etiology is cardiac. In a large published series, transcatheter closure was successful in 97% of patients, with oxygen saturation improving immediately from 84.6% to 95.1% (p<0.001) and dyspnea improving from New York Heart Association (NYHA) functional class 2.7 to class 1 (p<0.001) [6,7]. A single-center study reported that 64.8% of patients with POS experienced improved oxygen saturation after PFO closure [8]. However, closure is less effective when pulmonary disease is the primary etiology [8]. Given our patient's history of significant tobacco exposure and mild obstructive lung disease on pulmonary function testing, careful documentation of positional hypoxemia will be important to confirm that the intracardiac shunt is the primary driver of her symptoms.

This case has limitations. Formal orthodeoxia testing with arterial blood gas analysis in the supine and upright positions has not yet been performed. She is to undergo a cardiac MRI to look for anomalous pulmonary veins. The diagnosis of POS remains presumptive until positional desaturation is documented. Additionally, JAK2 mutation testing was not performed, though the normal erythropoietin level and absence of other myeloproliferative features make polycythemia vera unlikely.

## Conclusions

POS should be considered in patients presenting with unexplained erythrocytosis and dyspnea, particularly when symptoms have a positional component. The triad of positional dyspnea, intracardiac communication, and secondary erythrocytosis with normal EPO should prompt evaluation for POS. Early recognition is important, as percutaneous closure of the interatrial communication can be curative, with resolution of both respiratory symptoms and hematologic abnormalities. This case highlights the value of multidisciplinary collaboration between hematology, cardiology, and pulmonology in diagnosing complex presentations and underscores the importance of systematic screening for cardiopulmonary causes in patients with unexplained erythrocytosis.
